# Primary tumor resection in stage IV unresectable colorectal cancer: what has changed?

**DOI:** 10.1007/s12032-017-1047-6

**Published:** 2017-10-30

**Authors:** Michał Pędziwiatr, Magdalena Mizera, Jan Witowski, Piotr Major, Grzegorz Torbicz, Natalia Gajewska, Andrzej Budzyński

**Affiliations:** 1 2nd Department of General Surgery, Jagiellonian University Medical College, Kopernika 21, Kraków, Poland; 2 Centre for Research, Training and Innovation and Surgery (CERTAIN Surgery), Kraków, Poland

**Keywords:** Stage IV colorectal cancer, Unresectable colorectal cancer, Primary tumor resection, Decision making

## Abstract

Most current guidelines do not recommend primary tumor resection in stage IV unresectable colorectal cancer. Rapid chemotherapy development over the last decade has substantially changed the decision making. However, results of recently published trials and meta-analyses suggest that primary tumor resection may in fact be beneficial, principally in terms of prolonged survival. Additional factors, such as use of minimally invasive approach or protocols of enhanced recovery after surgery, affect clinical outcomes as well, but are often neglected when discussing the state of the art in this area. There are still no randomized studies determining the legitimacy of upfront surgery in asymptomatic patients. Also, quality of life also plays an important role in choosing appropriate treatment. Having said that, there is no data that would prove whether primary tumor resection has an advantage on that issue. With all the uncertainty, currently decision making in unresectable stage IV colorectal cancer is primarily up to clinicians’ knowledge, common sense and patients’ preferences.

## Introduction

Colorectal cancer (CRC) is the most common gastrointestinal cancer worldwide, the third most commonly diagnosed malignancy and fourth cause of deaths due to neoplasms [1]. Men are more susceptible to develop CRC than women [1, 2]. It is estimated that in 2017 135,430 new patients will be diagnosed with CRC worldwide [3]. Five-year survival rates depend mostly on the stage of cancer. The localized disease is found in less than 40% of primary diagnoses, where 5-year survival rates reach nearly 90%. When the disease is regionally advanced (spread to regional lymph nodes), over 70% of patients will survive 5 years. In stage IV with distant metastases, which is found relatively often (more than 20% of primary diagnoses), overall 5-year survival is less than 14% (or even less in rectal cancer cases) [3]. Long-term survival exceeding 5 years in disease with distant metastases was 9% when treated only with palliative chemotherapy and 36% when surgical and systemic treatment was combined [4, 5]. Therefore, aggressive surgical treatment has been proved to bring advantage in all patients eligible for radical metastasectomy [6, 7].

In 2013, a consensus of multidisciplinary management of CRC has been achieved and EURECCA guidelines have been published. Together with 2016 ESMO guidelines and ASCRS guidelines, they provide a standard of colorectal cancer treatment [8–10]. The fundamentals of treatment have been similar for years. Treatment should be provided by a multidisciplinary team of surgeons, medical oncologists, pathologists, radiation oncologists and radiologists. At the very beginning, it is crucial to assess the resectability criteria [9]. When considering management of CRC, localization, size of the primary tumor and all metastases as well as patient’s general health condition should be considered.

If the CRC stage allows radical resection of the primary tumor, with no doubt, R0 surgery is an upfront treatment option. In majority of rectal cancer cases neoadjuvant radiotherapy or radio-chemotherapy should be pondered [8]. Initially unresectable disease (both primary tumor and/or metastases) may become resectable after applying systemic treatment. This procedure is known as conversion therapy and has been proved to maintain better long-term outcomes than chemotherapy alone [9, 11].

The group of metastatic patients is highly heterogeneous, and each case should be considered separately. In 2016, Franko et al. [12] published an article in which they prove that localization of metastases influences survival rates. Patients with only lung metastases have significantly better overall survival rates than those with liver metastases, while patients with peritoneal spread have the worst prognosis. The analysis also showed that patients with one-organ non-peritoneal metastases have the best prognosis and the worst concerns patients with non-isolated peritoneal metastases [12]. Metastasectomy (as long as R0 resection can be achieved) has been performed to cure metastatic disease in the liver and lung more frequently for colorectal cancer metastases than for any other malignant neoplasms [13]. Whether those metastases are synchronous or metachronous is also of great importance, because metachronous hepatic and pulmonary resection has significantly better survival compared to the synchronous [14].

Nevertheless, combining both extensive and multivisceral surgical resections with preoperative or perioperative systemic drugs can lengthen survival in the majority of patients and even permanent cure in some cases is possible [15].

Local recurrences (more frequent when the primary tumor origins from rectum, where achieving clear resection margins is more complex) reduced significantly since the introduction of total mesorectal excision and complete mesocolic excision [16, 17]. Extended resections or pelvic exenterations often require expertise from other specialties (for example, urology, orthopedic surgery, plastic surgery or gynecology) [18]. Managing with recurrent disease is a challenge for today surgeons; however, it is inherent and inevitable part of advanced cancer treatment. This leads to a conclusion that all attempts should be undertaken to achieve R0 resections, and surgical intervention should be pondered as it affects patients’ survival.

## Treatment of unresectable disease

In approximately 65% of patients with stage IV CRC, a radical surgery is not possible [19]. In those cases, the issue that comes to the forefront is whether the disease is symptomatic or not. Up to 6% of stage IV CRC patients represent emergency conditions, such as active or occult bleeding, perforation or mechanical bowel obstruction [19, 20]. These individuals for obvious reasons require surgical intervention. This may simply prolong survival or improve patients’ general health condition and enable subsequent palliative systemic treatment. Moreover, it also impacts patients’ quality of life [9].

But what about resection in asymptomatic unresectable patients? Recent guidelines—EURECCA, ESMO and ASCRS—advise not to perform primary tumor resection (PTR) in asymptomatic cases with metastases that are beyond possibilities of surgical treatment [8–10]. Many points in the currently effective guidelines have been formed based on a 2012 Cochrane systematic review by Cirocchi et al. [19] in which they found that resection of the primary tumor in unresectable stage IV asymptomatic patients (who are managed with chemo- or radiotherapy) does not influence overall survival. Also, although resection does reduce the risk of tumor-related complications, these are relatively uncommon making them less clinically important. In addition, there is no point in delaying systemic treatment administration. These conclusions were based on the analysis of 7 studies (1086 patients; 722 patients treated with primary tumor resection and 364 patients managed first with chemotherapy and/or radiotherapy). However, the risk of selection, channeling or performance bias is significant, because none of those articles was based on a randomized trial. Studied groups differed in the type of surgery and were not homogenous in the patients’ recruitment. In the group where resection was performed, the size and localization of metastases was more favorable. What is more, authors agree that there still is enough doubt with regard to the published literature to justify further clinical trials in this area [19].

Chemotherapy development over the years also had a significant impact on decision making. Population-based study by Hu et al. [21] demonstrated that although primary tumor resection rate in stage IV CRC patients has been decreasing since 1990s, median relative survival rate has increased with annual percentage growth of 2.18% in 1988–2001 and 5.43% in 2001–2009. This study compared 64,157 patients with stage IV colorectal cancer using data from national cancer registry. Authors suggest that these findings strongly coincide with advantages in chemotherapeutic agents that started after 2000. Recent studies confirm this shift in decision making, demonstrating the use of newer chemotherapeutic agents and that these changes are associated with improved survival [22]. Modern, triple-drug chemotherapy has also been associated with reduced need of palliative surgery when compared to conventional 5-fluorouracil-based chemotherapy (20% vs. 10%) [23]. Chemotherapy is even more convincing when comparing overall survival in patients treated with modern systemic agents [24] in comparison with old fluorouracil and leukovorin approach [25] (over 29 vs. 4.3 months).

Another question is: will delaying the chemotherapy in order to perform a surgery influence the survival? The more demanding and complicated surgery, the more frequent occurrence of serious complications. Postponing the introduction of systemic treatment for eight or even 4 weeks significantly decreased both overall and disease-free survival rates [26, 27].

## Latest literature reviews and meta-analyses supporting primary tumor resection

In few recent years, there have been a few reviews that could change current perspective on decision making in stage IV CRC. Some papers report survival benefit when patients underwent primary tumor resections (PTR); however, these are cohort studies that use propensity score-matched approach, which may be a limitation [28]. Meta-analysis by Clancy et al. [29] showed longer OS when primary tumor resection approach was used. This meta-analysis included 21 papers, with over 44 thousand patients, that compared resection and chemotherapy in colorectal cancer with unresectable metastases. Most of included papers were retrospective reviews, while only two were cohort studies [30, 31]. As a result, PTR was associated with lower mortality risk (OR 0.28; 95% CI 0.165–0.474) and 6.4 months longer median overall survival.

A pooled post hoc analysis by Feo et al. [32] of four randomized trials confirms this correlation—PTR is in fact associated with longer OS in multivariate analysis (median 19.2 vs. 13.3 months—RCTs from 1997 to 2008). All authors are very careful when commenting positive results. Pezold et al. [33] did a systematic review of papers published between 2010 and 2015, and, although, there are few papers showing benefit with PTR, authors say that retrospective studies with multiple limitations and possible bias are not enough to say straightforward whether this is the correct approach. This is because PTR is often chosen as a treatment of choice for patients with tumors smaller and localized in colon.

It is worth mentioning that there are studies showing sometimes systemic treatment is only effective when following primary tumor resection. Ghiringhelli et al. [34] showed that the survival benefit of bevacizumab in 409 patients with metastatic CRC was only present in patients who underwent PTR previously.

The topic of performing randomized controlled trials has been recently brought back due to publication of new trial results. A study by Faron et al. analyzed data on 810 patients (478 in resection vs. 332 in non-resection group) from four first-line chemotherapy randomized trials. The analysis showed that primary tumor resection was independently associated with better overall and progression-free survival [35]. Ahmed et al. in retrospective cohort study compared patients who underwent surgical resection of primary tumor and received chemotherapy with patients treated with chemotherapy alone. Median overall survival in these groups was 18.3 and 8.4 months, respectively [36]. The analysis of 11,716 chemotherapy-treated stage IV colorectal cancer patients in California Cancer Registry was performed and published in 2014 by Tsang et al. Study showed that surgery was linked to higher median overall and colorectal cancer-specific survival (21 vs. 10 months and 22 vs. 12 months, respectively—in surgery vs. no surgery groups) [37].

Many reviews in the field underline the need of quality of life data [38, 39] which has been neglected by authors of previously conducted studies. Fortunately, some randomized controlled trials currently running have quality of life as their secondary outcome, including CAIRO4 study (NCT01606098), Spanish CCRe-IV study (NCT02015923) and GRECCAR8 study (NCT02314182).

## Surgical techniques in primary tumor resections

Considering the legitimacy of removing the primary tumor, we have to keep in mind that the incidence of surgical complications is high. What is more, there are significant differences in the difficulty of rectum versus colon procedures, which is reflected in the higher complication risk, longer length of hospital stay and slower recovery [40].

Although the rate of complications is comparable, their severity is different. Rectal resections are more for example commonly associated with higher-grade complications such as anastomotic leakage. It is of a great significance because the recovery after seriously complicated colorectal resection can affect the introduction of palliative chemotherapy which may have impact on survival. Therefore, in majority of cases even though primary tumor resection is performed, patients end up with colostomy rather than primary anastomosis. This obviously influences their quality of life. Accordingly, the decision about rectal resection has to be carefully discussed with the patient. Another question, besides whether to resect the tumor, is what surgical approach should be chosen. Similarly, there are no clear answers. From the point of view of oncologic outcomes, most recent meta-analyses show that laparoscopic approach is not inferior to open surgery both short term and long term [41]. However, this aspect seems irrelevant in patients with metastatic disease in whom R0 resection is not possible. Also, there are benefits connected directly to using minimally invasive approach. It has been proven multiple times that laparoscopy results in less surgical trauma, quicker recovery and—most importantly—much lower complications rate [42, 43]. All of this is especially important since primary tumor resection leads to approximately 5 weeks of delay in chemotherapy initiation [44], so approaches that allow its earlier start should be more beneficial. Some authors point out that—as a general rule—minimally invasive techniques should be used when available [45].

To additionally improve postoperative course, it is suggested to implement protocols of enhanced recovery after surgery (ERAS). Studies show that patients that follow ERAS protocol after colorectal surgery have lower complication rates and shorter length of hospital stay [46]. What is important in this case, it has been proven that ERAS protocol is also feasible for patients with stage IV colorectal cancer and there are no significant differences in morbidity, length of stay or readmissions rate between stages I–III and stage IV patients [47, 48].

## How would randomized controlled trials change our decision making?

Although the discussion about this specific problem has been going on for several years now, there are still no randomized controlled trials to give any sort of quantitative results that could decide which approach is dominant. There have been several attempts to conduct such studies, and some of them are currently running; however, it is relatively difficult to recruit patients to appropriate groups, which has been proved in example by previously designed trials that failed to do so (NCT01086618, NCT01978249—termination by problems with enrollment).

Difficulty of randomization is attempted to be by-passed by running cohort studies with propensity score matching, which—although being a fair replacement—has some drawbacks. Some may ask whether randomized controlled trials would change our approach to making decisions in this field. One thing that might be crucial is the fact that currently running studies are examining quality of life as their secondary outcome. This has been neglected previously, which resulted in this discussion.

There is a need for advanced prediction tools that would assist multidisciplinary teams in reaching treatment consensus. Previously, predictive models that have been created focused on hepatic resection for metastatic colorectal cancer [49–51], and only recently a model for predicting recurrence and survival in patients undergoing concurrent curative resection has been described [52]. Another aspect that might lead us into personalized, predictive medicine is utilizing knowledge about gene mutations, metabolic pathways and their impact on colorectal cancer development [53]. It has been shown that there are differences in, i.e., BRAF gene mutations between early- and late-stage patients [54, 55]. That information, along with advanced bioinformatics analysis, could help in creating accurate predictive models [56].

The problem of quality of life (QOL) improvement or deterioration is an underestimated but immensely important issue. Although QOL correction is one of the main concerns of palliative treatment, when compared with survival it is pushed to the background. There is still lack of trials assessing QOL in advanced cancer patients. In our opinion, the decision whether to ultimately exclude patient from surgical treatment should be made after deep and full of understanding of each patient’s needs.

## Conclusions

Looking at the most recent meta-analyses, more and more authors point out that there is a trend toward performing primary tumor resections. Still, we are not able to say definitely where and when to choose it over chemotherapy upfront, especially without data on quality of life. Hopefully, results from currently running controlled randomized trials will somehow aid decision making in the future. For now, this choice is primarily up to surgeon’s experience, patients’ preferences and common sense.
